# Prediction of prognosis, immune infiltration, and personalized treatment of hepatocellular carcinoma by analysis of cuproptosis-related long noncoding RNAs and verification *in vitro*

**DOI:** 10.3389/fonc.2023.1159126

**Published:** 2023-09-08

**Authors:** Shanbao Li, Zhonglin Zhu, Jing Lu, Wanyue Cao, Fangbin Song, Cao Xiao, Peng Zhang, Zeping He, Junyong Weng, Junming Xu

**Affiliations:** ^1^ Department of General Surgery, Shanghai General Hospital, Shanghai Jiao Tong University School of Medicine, Shanghai, China; ^2^ Department of Colorectal Surgery, Fudan University Shanghai Cancer Center, Shanghai, China; ^3^ Department of General Surgery, Fudan University Huashan Hospital, Shanghai, China

**Keywords:** cuproptosis, lncRNAs, HCC, TME, immunotherapy

## Abstract

**Background:**

The correlations between cuproptosis and long noncoding RNAs (lncRNAs) with the tumor microenvironment (TME), immunotherapy, and some other characteristics of hepatocellular carcinoma (HCC) remain unclear.

**Methods:**

Sixteen cuproptosis regulators and 356 cuproptosis-related lncRNAs (CRLnc) were identified from 374 HCC profiles in The Cancer Genome Atlas (TCGA) database. Six differentially expressed CRLnc were selected, and a prognostic risk model based on the CRLnc signature (CRLncSig) was constructed. The prognostic power of the model was verified. Moreover, a cuproptosis-related gene cluster (CRGC) was generated based on six lncRNAs and differentially expressed genes. The relationship between immune cell infiltration in the TME, immunotherapy, CRLncSig, and CRGC was demonstrated through various algorithms, Tumor Immune Dysfunction and Exclusion (TIDE), tumor mutational burden (TMB), etc. Potential drugs and sensitivity to those agents were evaluated for the risk model. LncRNA AL158166.1 was selected and verified in HCC tissues and cell lines, the impact of its knockdown and overexpression in HCC cells was examined, and the copper (Cu) concentration and the cuproptosis-related gene expression were detected.

**Results:**

A CRLncSig prognostic risk model with good predictive ability was constructed. The low-risk group had a longer overall survival (OS), lower tumor purity, more extensive immune cell infiltration, higher immune score, enrichment in immune-activated pathways, and more positive response to immunotherapy versus the high-risk group. CRGC-B exhibited the best OS and the lowest tumor stage; the immune cell infiltration analysis was similar to the low-risk group in CRLncSig. CRGC-B belonged to the “immune-high” group of the TME. The low-risk group had a higher TIDE score and susceptibility to antitumor drugs. The lncRNA AL158166.1 had the highest hazard ratio. The levels of AL158166.1 were higher in HCC tissues versus healthy tissues. Knockdown of AL158166.1 could lead to an increase in intracellular Cu concentration, induce DLAT low expression, and inhibit the proliferation and migration of HCC cells, whereas overexpression of AL158166.1 exerted the reverse effect.

**Conclusion:**

Overall, a new CRLncSig prognostic risk model and a cuproptosis-related molecular signature were constructed and evaluated. The model and signature were associated with the prognosis, immune infiltration, and immunotherapy of HCC. Inhibiting the lncRNA AL158166.1 may induce cuproptosis and showed potential for the inhibition of tumors. Evaluation of the CRLnc, CRLncSig, and CRGC may enhance our understanding of the TME, determine the effectiveness of immunotherapy, and act as a marker for the prognosis of HCC.

## 1 Introduction

Cell death has been a research hotspot in the field of life science. A new type of programmed cell death induced by copper (Cu), termed “cuproptosis,” was recently discovered. Excess intracellular Cu is related to mitochondrial metabolism, leading to proteotoxic stress and inducing cuproptosis (1, 2). The levels of Cu are higher in numerous types of cancer compared with healthy tissues. Cu accumulation is closely associated with angiogenesis and metastasis in cancer, including colorectal, pancreatic, breast, and liver (3–6). Hepatocyte apoptosis and mitochondrial oxidative damage are thought to be the mechanisms involved in Cu-induced hepatocyte injury. ATPase copper transporting beta (ATP7B) gene mutation induces a Cu secretion disorder of the bile duct and the accumulation of Cu in the liver. This leads to hepatocyte apoptosis and mitochondrial oxidative injury and the occurrence of hepatocellular carcinoma (HCC) (7). Cu could activate some angiogenic factors, including vascular endothelial growth factor (VEGF) and fibroblast growth factor (FGF) (8, 9). Moreover, the occurrence, development, and distant metastasis of HCC are closely related to tumor angiogenesis; VEGF plays a critical role in this process (10). However, the relationship between cuproptosis and cancer processes remains unclear.

Long noncoding RNAs (lncRNAs) perform mRNA-like functions, such as splicing, polyadenylation, and compiling (11). A growing body of evidence shows that lncRNAs are involved in the tumorigenesis of HCC. Numerous lncRNAs are maladjusted in HCC and participate in the cancer phenotype through binding with RNA, DNA, and proteins or encoding small peptides. These interactions result in continuous cell proliferation, thereby promoting tumor angiogenesis, evasion of apoptosis, etc. (12–14). LncRNAs can adjust the immune response and liver regeneration, serving as modulators of the liver immune microenvironment (15). Dysregulation of lncRNAs has been associated with chronic hepatitis and liver outgrowth, ultimately leading to the occurrence and progression of HCC (16). Epigenetic alterations, such as DNA methylation or histone modification, change the expression of lncRNA genes, thus promoting or inhibiting the progression of HCC (16, 17). Oncogenic transcriptional factors/cofactors, such as MYC, Yes-associated protein (YAP), and catenin beta (CTNNB), are overexpressed in HCC and promote the progression of HCC. For example, it has been shown that the MYC-regulated lncRNA LINC00176 is highly expressed in HCC, and its inhibition induces necroptosis (18). Moreover, lncRNAs change the cancer phenotypes by regulating microRNAs (miRNAs) and messenger RNAs (mRNAs) (19). Cuproptosis is currently attracting considerable research attention, and the role of cuproptosis-related lncRNAs (CRLnc) in HCC warrants further investigation.

The liver is an immune privileged organ, and HCC is a heterogeneous cancer with different etiologies, mutation profiles, and immune microenvironment; HCC could be immunogenic (20). Moreover, numerous immunotherapies for HCC have been investigated in clinical and preclinical studies in recent years (21, 22). The tumor microenvironment (TME) is composed of HCC cells, extracellular matrix, vascular endothelial cells, and infiltrating immune and stromal cells (23). The cancer-related fibroblasts in the extracellular matrix, vascular endothelial cells, and infiltrating immune and stromal cells promote tumor progression and regulate the efficacy of cancer treatments (e.g., immune checkpoint molecules on their surface and therapies directed toward the tumor-associated stroma and vasculature) (24, 25). HCC is a highly vascularized type of tumor that exploits angiogenesis for its growth and dissemination. This characteristic renders vascular-targeting approaches, namely, tyrosine kinase inhibition, appealing for the treatment of HCC; such agents include anti-vascular endothelial growth factor receptor (anti-VEGFR) and VEGF inhibitors (20, 26). Immune checkpoint inhibitors (ICIs), such as cytotoxic T lymphocyte-associated protein 4 (CTLA4), programmed cell death 1 (PDCD1), and other inhibitors, have also been utilized in the second-line treatment of HCC (27, 28). Some lncRNAs have been associated with the immune regulation of T cells, dendritic cells, and macrophages (29, 30). For example, lnc-epidermal growth factor receptor (lnc-EGFR) is a potential enhancer of EGFR in T cells, which induces and promotes the immunosuppression of HCC (29). Therefore, the role of lncRNAs in the TME and immunomodulation should be further investigated.

## 2 Materials and methods

### 2.1 Dataset source and RNA data

In this study, RNA-sequencing data of 374 HCC samples were extracted from The Cancer Genome Atlas (TCGA) database; 356 cuproptosis-related lncRNAs were retrieved, and six of those were selected. **Supplementary Figure S1** presents the workflow of this study. RNA-sequencing data, somatic mutation data, and clinical annotation of HCC were obtained from TCGA database. The RNA profiles (50 healthy and 374 cancer) were merged based on the fragments per kilobase million format (31).

### 2.2 Screening and identification of CRLnc

Sixteen cuproptosis-related genes (CRGs) were identified from literature on the cuproptosis-related cell death pathway, including seven upregulators (*FDX1, DLAT, LIPT1, PDHA1, LIAS, DLD,* and *PDHB*), three downregulators (*MTF1, GLS,* and *CDKN2A*), three carriers (*SLC31A1, ATP7A,* and *ATP7B*), and three enzymes (*DBT, GCSH,* and *DLST*) (1, 2). Pearson correlation analysis was performed to determine the co-expressed lncRNAs associated with those 16 CRGs based on the following criteria: Pearson correlation coefficient R > 0.4 and *P*< 0.001. Next, screening for differential expression of lncRNAs between healthy and cancer samples was conducted using log2 fold-change >1 and a false discovery rate<0.05 by “Limma” package (version 3.17).

### 2.3 Construction of a prognostic model based on the CRLncSig

Firstly, patients with HCC were randomly classified into the training or test group. Secondly, prognosis-related lncRNAs were selected by univariate Cox proportional hazards regression analysis. Thirdly, least absolute shrinkage and selection operator (LASSO) analysis was performed to optimize the selected lncRNAs. Finally, the best prognostic model was obtained through multivariate Cox proportional hazards regression analysis. The risk score was calculated as follows:


$$\text{Risk score=}\sum_{i=1}^{n}coef\left(lncRNA_{i}\right)\;*\;exp\left(lncRNA_{i}\right)$$

where *coef(lncRNA_i_)* and *exp(lncRNA_i_)* represent the coefficient and level of each lncRNA, respectively.

### 2.4 Construction of a nomogram and evaluation of the prognostic model

HCC samples were divided into the high- and low-risk groups based on the median risk score. In the training, test, and all-cohorts groups, the predictive capability of the prognostic model was evaluated using the Kaplan–Meier (KM) curve, receiver operating characteristic (ROC), univariate and multivariate Cox proportional hazards regression analyses, concordance index (C-index), principal component analysis (PCA), and area under the ROC curve (AUC) analysis. A nomogram was established to predict overall survival (OS) rates by combining the risk score with age, sex, and disease stage.

### 2.5 Unsupervised consensus gene clustering for CRLnc

Based on the CRLnc and prognosis-related lncRNAs, the R package “ConsensusClusterPlus” was used to conduct unsupervised consensus gene cluster analysis, which divided patients with HCC into three different subtypes (CRGC-A, CRGC-B, and CRGC-C).

### 2.6 Correlations of CRLnc Clusters with prognosis and cell infiltration in the TME

We evaluated the clinical value of the three CRLnc clusters (CRLncCs) using the KM curves. The tumor purity, estimation of stromal and immune cells in malignant tumor tissues using expression data (ESTIMATE) score, immune score, and stromal score were determined through the ESTIMATE algorithm. To explore cell infiltration in the TME, the proportions of immune cell subsets were calculated using the CIBERSORT algorithm. A single-sample gene-set enrichment analysis was performed to evaluate the enrichment scores of immune cell infiltration and immune function in the TME (32).

### 2.7 Correlations of CRLncSig and cuproptosis-related gene cluster with tumor mutational burden, chemotherapy, and immunotherapy in HCC

The somatic mutation profiles were obtained from TCGA; significantly mutated genes were identified and the tumor mutational burden (TMB) was determined using the “maftools” package. The efficiency of antitumor drugs was evaluated, and the half maximal inhibitory concentration (IC_50_) of 251 chemotherapy drugs (e.g., capecitabine, AKT inhibitor, and oxaliplatin) was calculated by the “pRRophetic” package. Tumor Immune Dysfunction and Exclusion (TIDE) was used to identify biomarkers for the prediction of response to immunotherapy in patients with HCC based on their pretreatment profiles (33). Data on the immune cell proportion score were obtained from The Cancer Immunome Atlas. Therapeutic differences of immunotherapy drugs (i.e., anti-PDCD1 and anti-CTLA4 antibodies) in patients with HCC were evaluated in two CRLncSig groups.

### 2.8 Tissue specimens and cell culture

Thirty pairs of primary HCC and healthy tissues were obtained from January 2020 to December 2021 at Shanghai General Hospital (Shanghai, China). Preoperatively, these patients had not received treatment. This research was approved by the ethics review committee of Shanghai General Hospital, and informed consent was provided by all patients. The Lo2 cell line was provided by Lu Jing (Shanghai General Hospital, Shanghai Jiao Tong University School of Medicine). The HCC cell lines (i.e., LM3, HepG2, Huh-7, PLC, and MHCC-97H) were provided by Dr. Zhang Peng and Xiao Chao (Fudan University Huashan Hospital). These cell lines were tested by short tandem repeat and authenticated in 2021 and 2020, respectively. The cells were cultured in Dulbecco’s modified Eagle’s medium (Invitrogen, USA) containing 10% fetal bovine serum (Gibco, USA) and 1% penicillin-streptomycin (Gibco, USA).

### 2.9 Primers, short hairpin RNAs, overexpression, and cell transfection

The primers used in quantitative reverse transcription-polymerase chain reaction (qRT-PCR) were as follows:

**Table:** 

Target genes	Primers
AL158166.1	Forward:5′-TATGTGCGTGGGAACTTGCT-3′
	Reverse: 5′- GCAGAATGCTTTGCCGTGAA-3′
shRNA1	CTAGCCCTTCATTCATCAAAT
shRNA2	CTCAGGAGGTGCCTTCCTAAT
shRNA3	TCTGGAAGTCACTCTCCATTT
scramble shRNA	CCTAAGGTTAAGTCGCCCTCG
OV	AGCACCGGGAAGCAGGGGCCGAGCCTTCCCCGCTCTGTGCCTGGGGGCTCTGGGCACTGTCTACACCTGGGAGGTGGTCAGCATATGTGCGTGGGAACTTGCTGGAAGGAAGGAATGACCCTTCTAGCCATGGAACGGTGGTCATCCGTGAGCCACGGCCTCAGGAGGTGCCTTCCTAATGTGCAGGTGACGATTGCCTCTGAGCTCAGGCATGCAGCTTCTGGGATGCACTCTCCTTTCACGGCAAAGCATTCTGCATAGGAAAAACCGTCTCTGCGTCTCCAAGCTGTGGCCTAGCCCTTCATTCATCAAATGGGATACAACTTTCATTTCTTATTAAAATCTGGAAGTCACTCTCCATTTGTAAGAT
GAPDH	Forward: 5′-GTCTCCTCTGACTTCAACAGCG-3′ Reverse: 5′-ACCACCCTGTTGCTGTAGCCAA-3′

LM3 and PLC cells were transfected with AL158166.1 short hairpin RNA (shRNA) and overexpression (OV), respectively; the scramble plasmids were transfected using Lipofectamine 2000 (Invitrogen, USA).

### 2.10 qRT-PCR

Total RNA was extracted from tissues and cells using TRIzol (Sigma, USA). The synthesis of complementary DNA was performed using the HyperScript III 1st Strand cDNA Synthesis Kit (EnzyArtisan, China). LncRNA AL158166.1 expression levels were examined by qRT-PCR performed using the S6 Universal SYBR qPCR mix (EnzyArtisan, China). The expression was determined according to the 2^−ΔΔCt^ method, and the levels of transcripts were normalized to those of glyceraldehyde-3-phosphate dehydrogenase (GAPDH).

### 2.11 Scratch wound healing assay

Transfected LM3 and PLC cells by AL158166.1 shRNA, OV, and scramble plasmids were seeded in six-well plates until they reached approximately 80%–90% confluence. Subsequently, a wound was inflicted in the middle of the well using a pipette tip. Images were captured at 0 and 36 h after inflicting the wound. This experiment was repeated thrice.

### 2.12 Transwell migration assay

Transfected LM3 and PLC cells were seeded in the upper chamber of a Transwell assay (Corning, NY, USA) containing serum-free medium (200 μL); medium supplemented with 10% fetal bovine serum (600 μL) was placed in the lower chamber. The migrated cells were fixed and stained using methanol and crystal violet, respectively. The results were based on the analysis of 10 random fields.

### 2.13 Cell proliferation

Transfected LM3 and PLC cells were seeded in 96-well plates containing medium with 10% fetal bovine serum (3,000 cells per well). After cell culture for 24, 48, 72, and 96 h, Cell Counting Kit-8 (10 μL) reagent was added to each well, and the cells were incubated for another 2 h. Thereafter, the optical density was detected at 450 nm using a standard microplate reader.

### 2.14 Measurement of intracellular copper

Transfected LM3 and PLC cells were seeded in 6-cm plates containing medium with 10% fetal bovine serum. According to the manufacturer’s instructions, after cell culture for 24 h, cells were collected, resuspended, and ultrasonically degraded to detect intracellular Cu (Elabscience, Wuhan, China).

### 2.15 Immunoblot analysis

Proteins were extracted from liver cells, mixed with sodium dodecyl sulfate-polyacrylamide gel electrophoresis (SDS-PAGE) sample loading buffer (Cat. No.: P0015L, Beyotime Biotechnology, Shanghai, China), and transferred onto polyvinylidene fluoride (PVDF) membranes (Cat. No.: CCGL52TP1, Millipore, Billerica, MA, USA) after electrophoresis in a 4°C environment. Membranes were blocked in NcmBlot blocking buffer (Cat. No.: P30500, NCM Biotech, Suzhou, China) for 10 min and then incubated with primary antibodies at 4°C overnight. The primary antibodies included anti-DLAT antibody (Cat. No.: 13426-1-AP, 1:1,000, Proteintech, USA) and GAPDH monoclonal antibody (Cat. No.: 60004-1-Ig, 1:5,000, Proteintech, USA). The secondary antibodies included goat anti-rabbit IgG (Cat. No.: SA00001-2, 1:5,000, Proteintech, USA). Proteins were detected by enhanced chemiluminescent (ECL) reagent (Cat. No.: 180-501, Tanon, Shanghai, China).

### 2.16 Statistical analyses

For bioinformatics data, statistical analyses were conducted using the R 4.1.3 software (4.1.3 version, R studio, Boston, MA, USA). All data derived from basic research were analyzed with GraphPad Prism (version 8.0; GraphPad Software, San Diego, CA, USA). Student’s t-tests were applied to assess differences between two groups. All experiments were conducted independently and repeated three times. The *P* value<0.05 was set as the statistical significance. All data are presented as mean values ± SEM. The statistical details are all specified in the figure legends unless otherwise indicated.

## 3 Results

### 3.1 Screening and identification of CRLnc in HCC

**Figure 1:**
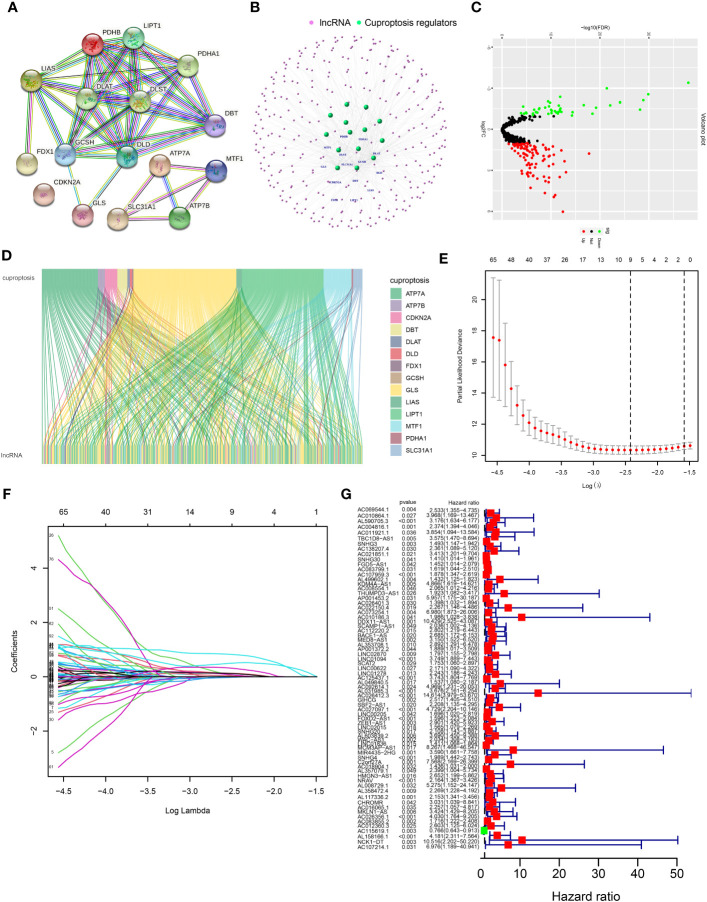
Network of lncRNA–mRNA co-expression and screening of cuproptosis-related lncRNAs in patients with HCC. **(A)** Volcano plot showing different expressions of lncRNAs between healthy and HCC cancer samples. Red, black, and green dots denote upregulated, unchanged, and downregulated, respectively. **(B)** A PPI network of 16 cuproptosis regulators was analyzed through the STRING database. **(C)** The lncRNA–mRNA co-expression network by R package “igraph.” **(D)** Sankey diagram of cuproptosis regulators and prognosis-related lncRNAs by R package “ggalluvial.” **(E)** Cross-validation in the LASSO model. **(F)** Forest diagram of the LASSO coefficient profile of prognosis-related lncRNAs. **(G)** Results of the univariate Cox proportional hazards regression analysis of prognosis-related lncRNAs. HCC, hepatocellular carcinoma; LASSO, least absolute shrinkage and selection operator; lncRNA, long noncoding RNA; PPI, protein–protein interaction; STRING, Search Tool for the Retrieval of Interacting Genes.

Sixteen CRGs were identified, including seven upregulators (FDX1, LIPT1, LIAS, DLD, DLAT, PDHA1, and PDHB), three downregulators (MTF1, GLS, and CDKN2A), three carriers (SLC31A1, ATP7A, and ATP7B), and three enzymes (DBT, GCSH, and DLST). The protein–protein interaction network and Search Tool for the Retrieval of Interacting Genes (STRING) database were utilized to show the interactions of these CRGs (**Figure 1A**; **Supplementary Table S1**). The co-expressed lncRNAs of these 16 CRGs were identified by Pearson correlation analysis (**Supplementary Table S2**). The co-expressed network of 356 CRLnc and mRNA is shown in **Figure 1B**. According to the selection criteria, there were 151 differentially expressed CRLnc (**Figure 1C**; **Supplementary Table S3**). The Sankey diagram was used to evaluate the 16 CRGs and co-expressed CRLnc (**Figure 1D**).

### 3.2 Construction and assessment of the CRLncSig prognostic model

The prognostic differentially expressed CRLnc was optimized by LASSO analysis to prevent overfitting (**Figures 1E, F** **)**. We identified lncRNAs related to the prognosis of HCC (**Figure 1G**). Six CRLnc (i.e., AL590705.3, AC107959.3, AL031985.3, GIHCG, LINC02015, and AL158166.1) were obtained by multivariate Cox proportional hazards regression model analysis (*P*< 0.05); the weighted coefficient is shown in **Supplementary Table S4**. Next, the CRLncSig prognostic model was constructed based on these six CRLnc. The heatmap showing correlations between the six CRLnc and 16 CRGs revealed that LIPT1, DLAT, MTF1, GLS, CDKN2A, and ATP7A were positively correlated with most lncRNAs (**Supplementary Figure S2A**).

**Figure 2:**
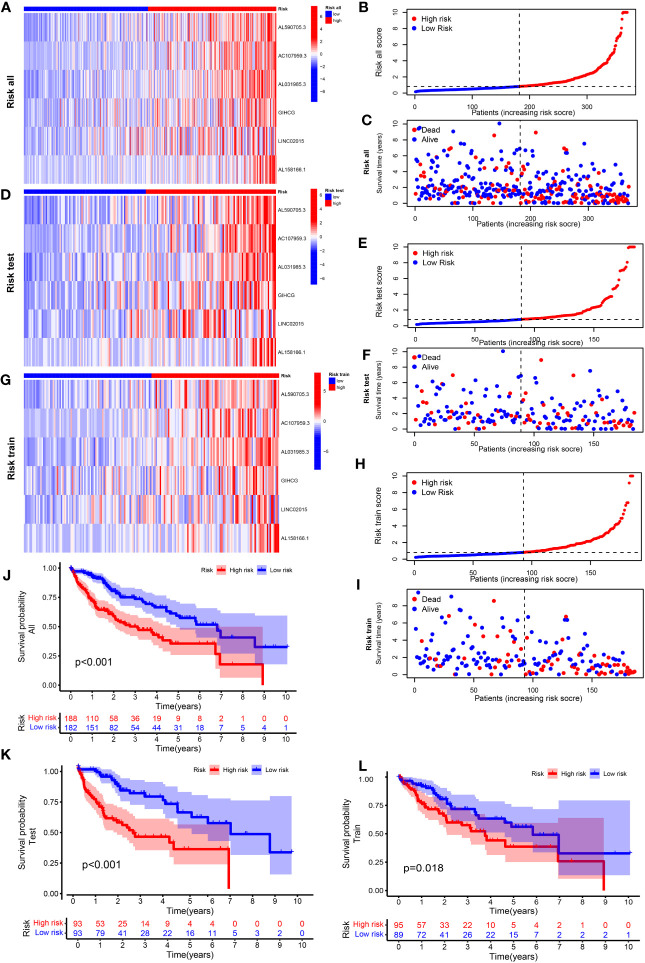
Construction of a cuproptosis-related prognostic model for patients with HCC. Heatmap of the expression of the six lncRNAs included in this model, scatterplot of risk score, and distribution of survival time and survival status of the CRLnc high- and low-risk groups in **(A–C)** all HCC samples; **(D–F)** test set HCC samples; and **(G–I)** training set HCC samples. **(J–L)** The Kaplan–Meier survival curves of overall survival between the CRLnc high- and low-risk groups in all samples (*P*< 0.001), training set (*P* = 0.018), and test set (*P*< 0.001). CRLnc, cuproptosis-related long noncoding RNAs; HCC, hepatocellular carcinoma; lncRNA, long noncoding RNA.

**Figure 3:**
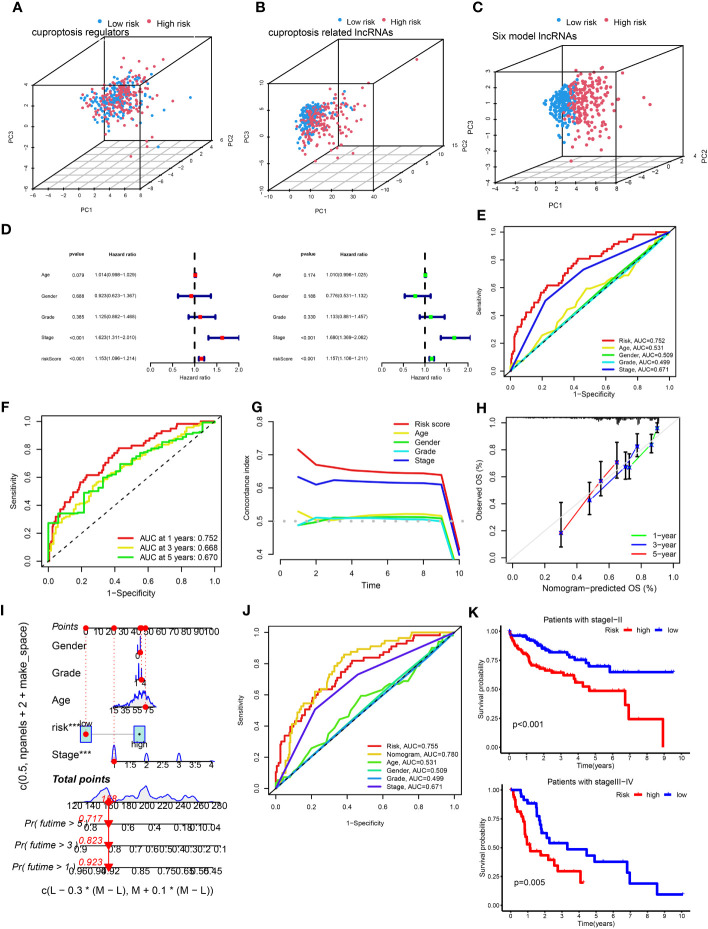
Assessment of the cuproptosis-related prognostic model and construction of a nomogram. **(A–C)** PCA of cuproptosis regulators, cuproptosis-related lncRNAs, and the six-lncRNA model according to the high and low risk of all patients with HCC. **(D)** Univariate and multivariate Cox proportional hazards regression analyses of overall survival in all HCC samples. **(E)** The 1-year ROC curves for risk score, age, sex, and stage in patients with HCC. **(F)** The 1-, 3-, and 5-year ROC curves for risk score in patients with HCC. **(G)** C-index curves for risk score, age, sex, and stage in patients with HCC. **(H)** Calibration curves predicting the survival probability at 1, 3, and 5 years. **(I)** The nomogram was constructed with gender, grade, age, risk score, and stage to predict the overall survival rate of 1, 3, and 5 years. **(J)** The nomogram was constructed using the risk score, age, sex, TNM stage, and stage to predict the overall survival rate at 1, 3, and 5 years. **(K)** Kaplan–Meier survival curves of overall survival between the high- and low-risk groups in patients with stage I–II (*P*< 0.001) and III–IV (*P* = 0.005) disease. C-index, concordance-index; HCC, hepatocellular carcinoma; lncRNA, long noncoding RNA; PCA, principal component analysis; ROC, receiver operating characteristic; TNM, tumor-node-metastasis.

**Supplementary Table S5** shows the groups analyzed in this study. The heatmap of lncRNA expression (**Figures 2A, D, F** **)**, scatterplot of the risk score (**Figures 2B, E, H** **)**, survival time (**Figures 2C, G, I** **)**, and survival curve (**Figures 2J–L**) were produced for the three groups. The high-risk group exhibited a worse OS compared with the low-risk group, indicating the effectiveness of the CRLncSig prognostic model. Exploring the differences between high-risk and low-risk groups, the PCA results showed that, unlike CRG (**Figure 3A**) and CRLnc (**Figure 3B**), the CRLnc risk score (**Figure 3C**) could most effectively distinguish the profiles. Next, we also investigated the role of the CRLnc risk score in the CRLncSig prognostic model and its value as an independent prognostic factor by the univariate and multivariate Cox regression analyses, respectively. The results showed that the tumor stage and risk score were significantly different (*P*< 0.001); their hazard ratios of risk score were 1.157 and 1.153, respectively (**Figures 3D**). These findings indicated the remarkable predictive efficiency of this model. The sensitivity and specificity of the CRLncSig prognostic model were evaluated using the ROC curve, time-dependent AUC, and PCA. The 1-year AUC values for disease stage, age, and sex were lower than that obtained for the risk score. The AUC values for OS at 1, 3, and 5 years were 0.752, 0.668, and 0.670, respectively; the C-index revealed a similar trend, indicating the greater predictive ability of the CRLnc risk score versus other parameters (**Figures 3E–G**).

### 3.3 Construction and verification of the nomogram

Firstly, the nomogram was constructed to predict OS at 1, 3, and 5 years (**Figure 3H**). Secondly, the calibration plots showed that the predictive efficiency of OS at 1, 3, and 5 years was consistent (**Figure 3I**). The 1-year AUC value of the nomogram was 0.78 (**Figure 3J**), indicating better predictive ability than 3- and 5-year AUC value. We also examined survival based on the CRLnc risk score for patients with stage I–II and stage III–IV HCC. The results showed that the high-risk group had a worse prognosis than the low-risk group (**Figure 3K**). In conclusion, the CRLnc risk score in the CRLncSig prognostic model exhibited excellent predictive ability for the prognosis of HCC.

### 3.4 Relationship of clinical characteristics and immune cell infiltration in the TME with the CRLnc risk score in HCC

**Figure 4:**
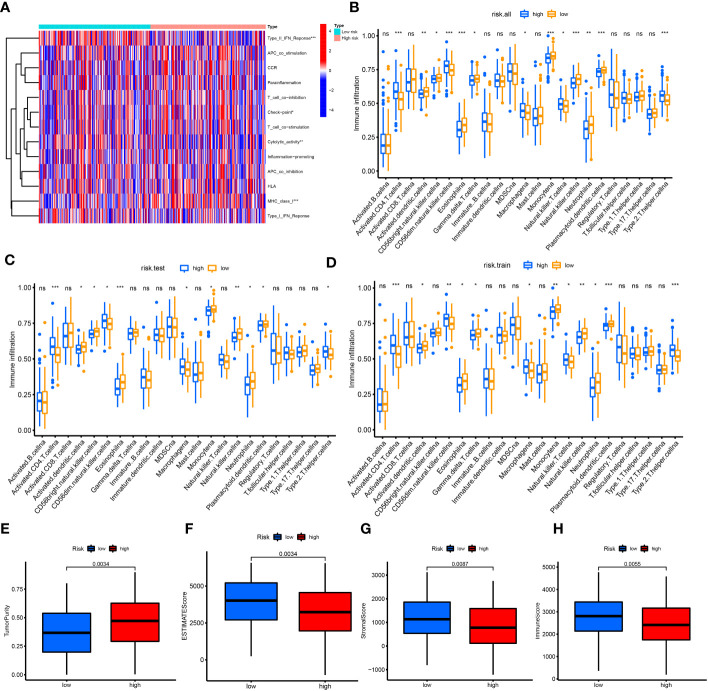
Correlation of immune cell infiltration with risk score. **(A)** Heatmap of immune reaction between high and low risk in all patients with HCC. **(B–D)** Enrichment of immune cells for all cohorts, test set, and training set in the high- and low-risk groups using the ssGSVA algorithm, respectively. **(E–H)** Tumor purity (*P* = 0.0034), immune score (*P* = 0.0055), stromal score (*P* = 0.0087), and ESTIMATE score (*P* = 0.0034) in the high- and low-risk groups using the ESTIMATE algorithm. *P< 0.05; **P< 0.01; ***P< 0.001. ESTIMATE, Estimation of STromal and Immune cells in MAlignant Tumor tissues using Expression data; HCC, hepatocellular carcinoma; ssGSVA, single-sample gene set variation analysis.

The heatmap showed that the low-risk group had significant differences in the process of interferon gamma (IFNG) response, cytolytic activity, and checkpoint, while the high-risk group exhibited changes in the process of major histocompatibility complex (MHC) class I response (**Figure 4A**). The results of immune cell enrichment analysis for the three groups showed that CD4+ T cells, type 2 T helper cells, and macrophages were enriched more in the high-risk group, while the CD8+ T cells, dendritic cells, natural killer cells, and natural killer T cells were more enriched in the low-risk group (**Figures 4B–D**). The TME in these groups was evaluated using the ESTIMATE algorithm; compared with the high-risk group, tumor purity was lower in the low-risk group, whereas the immune, stromal, and ESTIMATE scores were higher (**Figures 4E–H**). Therefore, the aforementioned clinicopathological characteristics and upper infiltration of the TME by immune cells were associated with a low CRLnc risk score.

### 3.5 Identification of cuproptosis-related signatures

**Figure 5:**
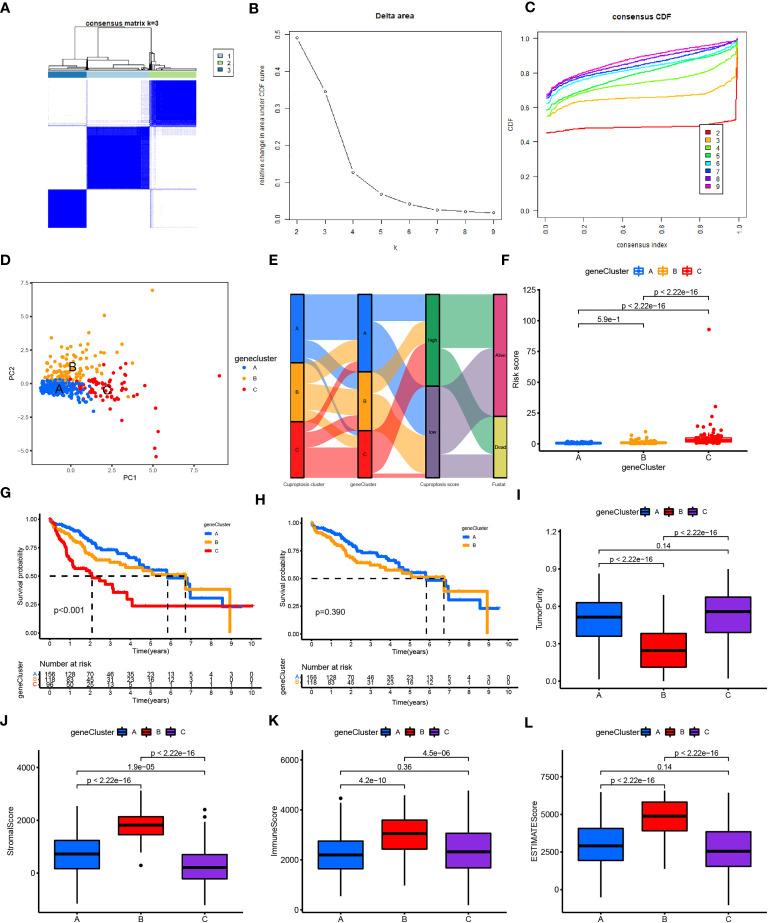
Construction and function annotation of cuproptosis-related gene clusters. **(A–C)** Consensus matrix heatmap defining three gene clusters (k = 3) and their correlation area. **(D)** PCA of three gene clusters to distinguish samples in TCGA HCC. **(E)** Alluvial diagram showing attribute changes from cuproptosis gene clusters to risk score and survival status. **(F)** Differences in risk scores between the three gene subtypes. **(G)** Survival analyses for the three gene clusters using Kaplan–Meier curves (*P*< 0.001). **(H)** Survival analyses for gene clusters A and B using Kaplan–Meier curves (*P*< 0.039). **(I–L)** Tumor purity (*P*< 0.001), immune score (*P*< 0.001), stromal score (*P*< 0.001), and ESTIMATE score (*P*< 0.001) in the three gene clusters according to the ESTIMATE algorithm. ESTIMATE, Estimation of STromal and Immune cells in MAlignant Tumor tissues using Expression data; HCC, hepatocellular carcinoma; PCA, principal component analysis; TCGA, The Cancer Genome Atlas.

The CRLncC-A/B/C (**Supplementary Figures S3A–S3C**) and CRGC-A/B/C (**Figures 5A–C**) were obtained using the consensus clustering algorithm (**Supplementary Tables S7, S8**). PCA results showed that, unlike CRLncC (**Supplementary Figure S3D**), the CRGC (**Figure 5D**) could effectively distinguish the profiles. The distribution of patients with HCC was described by three CRLncC, three CRGCs, and two CRLnc risk scores in the alluvial diagram (**Figure 5E**). The results showed that patient survival was associated with the low-risk group; most patients in CRGC-A/B and CRLncC-A were included in the low-risk group, while those in CRLncC-C and CRGC-C were mostly included in the high-risk group.

**Figure 6:**
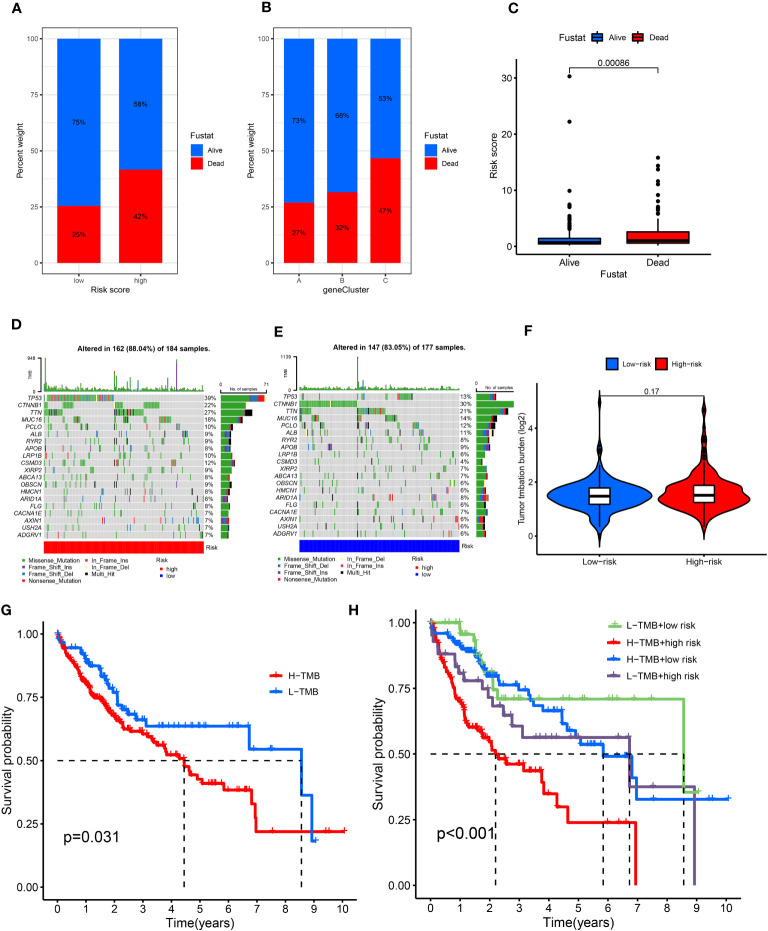
Relationship of cuproptosis patterns with tumor somatic mutation. **(A)** Patient survival status in the risk score groups. **(B)** Patient survival status in the three gene cluster groups. **(C)** Distribution of risk score in the alive and dead patients (*P* = 0.0086). **(D, E)** Waterfall plot of somatic mutation features established based on high- and low-risk scores. **(F)** Spearman correlation analysis of the risk score and TMB. **(G)** Survival analyses for the low- and high-TMB score patient groups using Kaplan–Meier curves (*P* = 0.031). **(H)** Survival analyses of the subgroups of patients stratified by both risk and TMB scores using Kaplan–Meier curves (*P*< 0.001). TMB, tumor mutational burden.

In addition, analysis of the future survival status of patients with HCC showed that the rates of living patients were better in the low-risk score group and CRGC-A/B versus the high-risk group and CRGC-C, respectively (**Figures 6A–C**). The relationship between the CRLnc risk score, CRLncC, and CRGC was evaluated using the Kruskal–Wallis test; cluster A/B of CRLncC and CRGC had the lowest risk score (**Figure 5F**; **Supplementary Figure S3E**). The KM curves showed significant differences in OS among the three CRGC and CRLncC. CRGC-A/B had a better OS than CRGC-C; however, there were no significant differences between CRGC-A and CRGC-B. Notably, differences between CRLncC-B and CRLncC-C were not statistically significant (**Figures 5G, H**; **Supplementary Figures S3F, S3G**). We further investigated the relationship between clinicopathological characteristics and CRLnc expression in the three CRGCs. The heatmap showed that the CRGC-C had the most advanced disease stage according to the Union for International Cancer Control and the best future survival state (**Supplementary Figure S2B**). The above results indicated that CRGC possesses better predictive ability versus CRLncC for the prognosis of HCC.

### 3.6 Immune cell infiltration of the TME in CRGC and CRLncC

We found that tumor purity was lower in CRGC-B (**Figure 5I**) and CRLncC-B (**Supplementary Figure S3H**) versus the other two clusters; however, the immune, stromal, and ESTIMATE scores in these two groups were the highest obtained in this analysis (**Figures 5J–L**; **Supplementary Figures S3I–S3K**).

CRGC-B showed a tumor-infiltrating lymphocyte environment that was more conducive to immunotherapy, including extensive infiltration by CD8+ T cells and CD4+ T cells, as well as enrichment of M1/M2 macrophages. In contrast, CRGC-C exhibited marked enrichment of M2 macrophages versus the other two groups (**Supplementary Figures S2C–S2E**). CRGC-B presented the lowest tumor purity, highest immune, stromal, and ESTIMATE scores, and enrichment of immune cells. These findings indicated that CRGC-B belonged to the “immune-high” phenotype; CRGC-C and CRGC-A represented the “immune-low” and “immune-mid” phenotypes, respectively. The trend shown by CRLncC for the evaluation of the TME was similar to that of CRGC. Nevertheless, this was not consistent with the trend observed for OS, and CRLncC could not predict the prognosis of HCC as effectively as CRGC. According to the analysis of future survival status of HCC patients, it is found that the living patients in low-risk score group and CRGC-A are better than those in high-risk and CRGC-B, C groups respectively (**Figures 6A–C**). Our results indicated that CRGC could predict the prognosis and characteristics of cell infiltration in the TME of HCC.

### 3.7 Investigation of the CRLnc risk model with TMB and clinical treatment

The top mutated genes were TP53, CTNNB1, TTN, and MUC16. TP53, MUC16, and TTN exhibited higher mutation frequencies in the high-risk group versus the low-risk group, while the mutation levels of CTNNB1 and TP53 showed an opposite trend (**Figures 6D, E** **)**. The results showed that there was no statistically significant relationship between the CRLnc risk score and TMB (*P* = 0.17) (**Figure 6F**). However, the KM curves showed obvious differences between the high- and low-TMB groups (*P* = 0.031); patients with high TMB were linked to a worse OS. The association of different TMB levels and CRLnc risk scores with OS was evaluated using the KM curves. The high CRLnc risk score and TMB group showed the worst OS, while the low CRLnc risk and TMB group exhibited the best OS (**Figures 6G, H** **)** versus the other groups.

**Figure 7:**
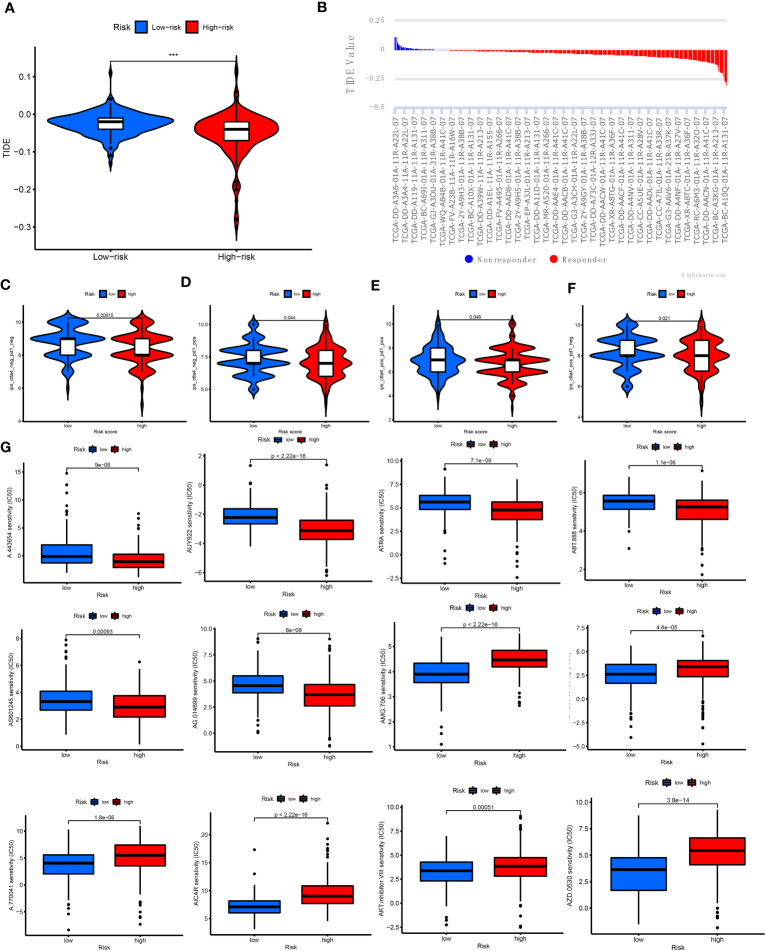
Relationship of cuproptosis patterns with clinical immunotherapy in patients with HCC. **(A)** Relative expression of TIDE in the high- and low-risk score groups. **(B)** TIDE predicted the degree of response to immunotherapy in patients with HCC. **(C–F)** Patients with response to PDCD1 and CTLA4 blockade immunotherapy in the low-risk score group. **(G)** IC_50_ of 12 antitumor drugs in the two risk groups. CTLA4, cytotoxic T lymphocyte-associated protein 4; HCC, hepatocellular carcinoma; IC_50_, half maximal inhibitory concentration; PDCD1, programmed cell death 1; TIDE, Tumor Immune Dysfunction and Exclusion.

Next, the relationship of immunotherapy with ICIs and the CRLnc risk score was examined. The TIDE showed that the low-risk group exhibited markedly better response to immunotherapy than the high-risk group (**Figures 7A, B** **)**. We further assessed the response to PDCD1 and CTLA4 blockade immunotherapy in the CRLnc risk score groups to examine the efficiency of ICIs. The results showed that the proportion of immune cells was greater in the low-risk group versus the high-risk group following anti-CLTA4 and anti-PDCD1 immunotherapy, indicating better response to immunotherapy in the former group (**Figures 7C–F**; **Supplementary Table S9**). Lastly, the results showed that the drugs (AKT inhibitor, mcl-1-specific inhibitor AZD5911 (AZD), BCL2 inhibitor ABT-263 (ABT), etc.) presented lower IC_50_ in the low-risk group than that in the high-risk group. This difference may assist in the evaluation of potential drugs for the treatment of HCC (**Figure 7G**).

### 3.8 LncRNA AL158166.1 was highly expressed in HCC and predicted prognosis

**Figure 8:**
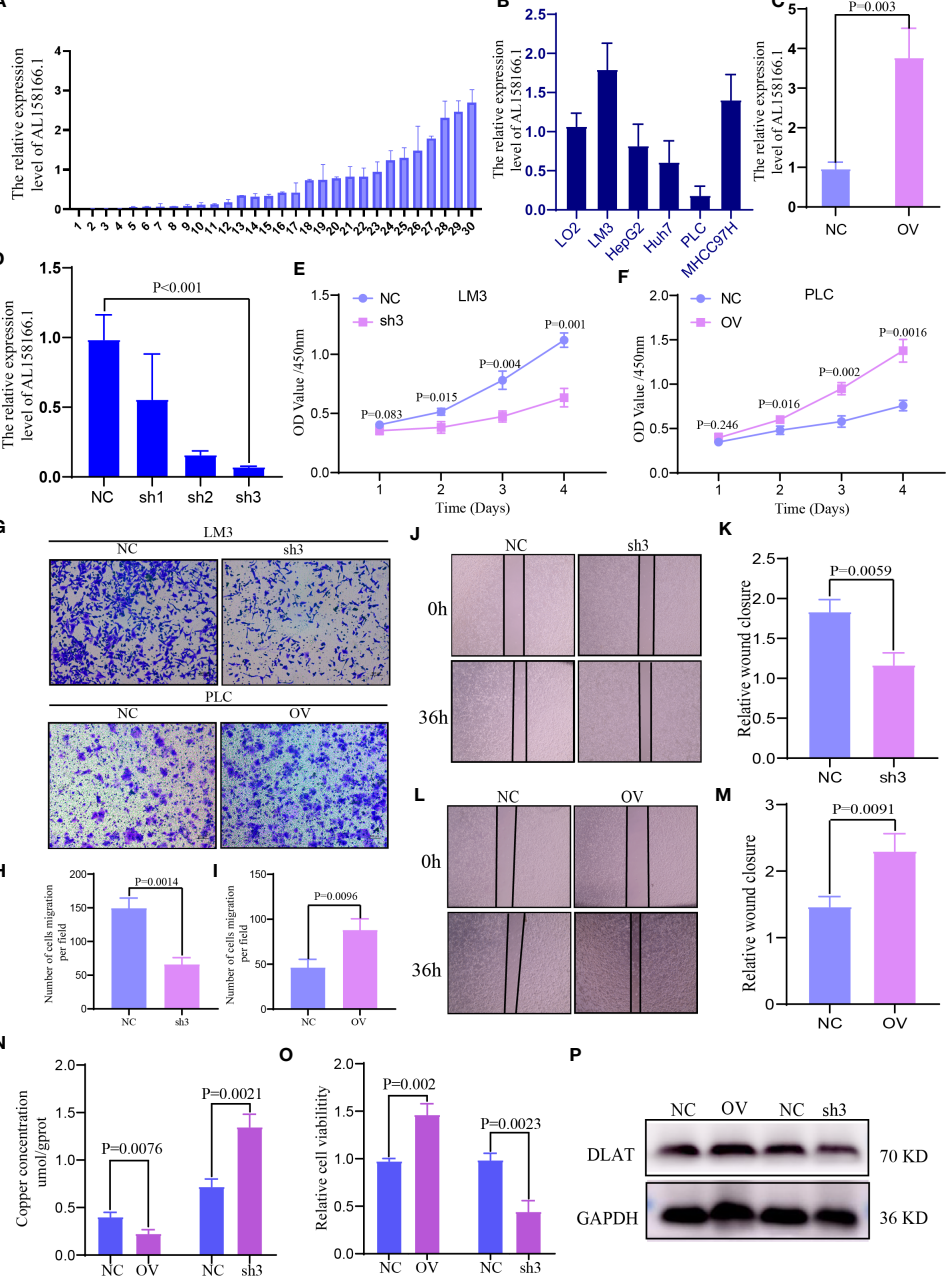
Expression and function of lncRNA AL158166 and it could induce cuproptosis *in vitro*. **(A)** Relative mRNA expression of lncRNA AL158166 in 30 pairs of HCC tissues and healthy adjacent tissues by qRT-PCR. **(B)** Relative expression of lncRNA AL158166 in Lo2 healthy liver cell line and other five HCC cell lines. **(C, D)** LM3 cells were transfected with lncRNA AL158166 silencing vectors, while PLC cells were transfected with overexpressing vectors; the knockdown and overexpression efficiencies were verified by qRT-PCR. Effects of lncRNA AL158166 knockdown and overexpression on cell proliferation and migration according to the CCK8 **(E, F)**, Transwell **(G–I)**, and wound healing assays **(J–M)**. The concentration of copper was detected after overexpression and knockdown in HCC cells **(N)**. Cell viability was detected after overexpression and knockdown in HCC cells **(O)**. Representative marker DLAT of cuproptosis was detected by WB after overexpression and knockdown in HCC cells **(P)**. Error bars represent the SEM. Original magnification: 100×. NC group: transfected with negative control lentivirus; sh3 group: transfected with shRNA3; OV group: transfected with overexpressing vector. CCK8, Cell Counting Kit-8; HCC, hepatocellular carcinoma; lncRNA, long noncoding RNA; NC, negative control; OV, overexpression; qRT-PCR, quantitative reverse transcription-polymerase chain reaction; DLAT, dihydrolipoamide S-acetyltransferase; SEM, standard error of the mean.

The lncRNA AL158166.1 exhibited the highest score among the six CRLnc (**Figure 1G**). Next, we detected the relative expression of AL158166.1 in 30 paired healthy and HCC tissues; AL158166.1 was highly expressed in most HCC tissues (**Figure 8A**).

### 3.9 High expression of lncRNA AL158166.1 reduced cell Cu accumulation and promoted the proliferation and migration of HCC cells

The highest expression of AL158166.1 was recorded in LM3 cells (**Figure 8B**). The efficiency of OV and knockdown was detected by qRT-PCR in PLC and LM3 cells; the shRNA3 sequence demonstrated the best knockdown efficiency (**Figures 8C, D** **)**. According to the results of Cell Counting Kit-8 assays, AL158166.1 knockdown inhibited the proliferation of LM3 cells (**Figure 8E**), while AL158166.1 OV promoted this phenomenon in PLC cells (**Figure 8F**). The Transwell migration and wound healing assays indicated that higher AL158166.1 expression increased the migratory ability of PLC cells, while lower AL158166.1 levels suppressed this phenomenon in LM3 cells (**Figures 8G–M**). We further evaluated whether knockdown and OV of lncRNA AL158166.1 led to changes in intracellular Cu accumulation. The levels of Cu increased after lncRNA AL158166.1 knockdown but decreased significantly after OV (**Figure 8N**), while the trend of cell viability was opposite (**Figure 8O**), which suggested that OV of lncRNA AL158166.1 could induce a decrease in Cu accumulation in HCC cells and promote HCC cell growth. Cu2+ could bind to thioacylated DLAT, inducing the isomerization of DLAT. The increase in insoluble DLAT leads to cytotoxicity and induces cell death (34). We verified and found that after knocking down lncRNA AL158166.1, the expression of DLAT decreased, while OV led to an increase in DLAT expression in HCC cells (**Figure 8P**). Collectively, these findings indicated that lncRNA AL158166.1 could induce the cuproptosis in HCC cells.

## 4 Discussion

Cu is an essential element for organisms and associated with numerous biological processes (35). Higher levels of Cu are found in tumor tissues versus healthy tissues, and the accumulation of Cu promotes the proliferation, angiogenesis, and metastasis of cancer (4, 36). Regulated cell death can be induced by some heavy metals. Cuproptosis is a newly discovered type of Cu-induced cellular death; the mechanism underlying this process may provide new ideas for the treatment of cancer (35). However, few studies have investigated the role of cuproptosis in the TME and ICIs of HCC. Hence, the regulation, transcription, and protein levels of CRGs should be elucidated in future studies.

In this study, we identified 16 CRGs from relevant literature and constructed a CRLncSig risk model composed of CRLnc to predict the prognosis and therapeutic effects of antitumor drugs in patients with HCC. In addition, clinicopathological characteristics and increased infiltration of the TME by immune cells were closely associated with the CRLnc low-risk score. Cancer-related lncRNAs are becoming a research hotspot in the fields of RNA biology and oncology. The OV of oncogenic lncRNAs and inhibition of cancer-suppressive lncRNAs are closely associated with aberrant transcription, thereby leading to the pathogenesis and progression of cancers. Such lncRNAs can bind to DNA, RNA, and proteins and subsequently change the expression, localization, stability, activity, etc., of their binding partners (14, 37, 38). LncRNAs have the potential to serve as biomarkers for HCC and could help in the prediction of response to treatment or the classification of tumors (12, 25).

Six CRLnc and prognosis-related lncRNAs were identified from TCGA. We next constructed a CRLncSig prognostic risk model based on these lncRNAs. The analyses demonstrated the outstanding predictive ability of this risk model. By constructing a nomogram, we found that a low CRLncSig risk score was closely related to positive clinical features and prognosis in patients with HCC. The upregulated and downregulated lncRNAs in HCC have been summarized in a previous review; most lncRNAs were significantly associated with clinicopathologic features, including the tumor size, focality, differentiation, invasion, tumor stage, metastasis, presence of cirrhosis, alpha fetoprotein levels, and hepatitis B virus infection status (19). LncRNAs have great potential in the diagnosis and treatment of HCC. Hence, further understanding of the complex mechanism underlying the effects of lncRNAs in the occurrence and metastasis of HCC could promote their use in clinical diagnosis and treatment.

Moreover, cuproptosis-related patterns (CRGC and CRLncC) were constructed. We found that CRGCs were closely correlated with clinicopathological features and infiltration of the TME by immune cells in HCC. The CRGCs characterized by immune activation and inhibition in the TME showed significantly different TMB, immune checkpoints, clinicopathological characteristics, and prognosis than other clusters. These findings indicated that the CRLncSig model may serve as a prognostic biomarker and predict patient responses to immunotherapy.

The blood supply in the liver is complex and the anatomy of this organ is special; consequently, this complexity complicates the TME and immunotherapy of HCC. Based on immune cell infiltration in the TME and the CRLnc risk score, HCC was classified into two subtypes (i.e., immune-high and immune-low) (39). The immune-high type was associated with a positive response to immunotherapy, as reflected by an increase in the number and activation of immune cells; in contrast, the immune-low type exhibited an opposite profile. The CRLnc low-risk group had lower tumor purity versus the high-risk group, increased stromal, immune, and ESTIMATE scores, and was involved in immune-activated functions and pathways. We further examined the efficiency of ICIs in HCC samples by TIDE and Time-lagged Independent Component Analysis (TICA) (33). The low CRLnc risk group showed a better immune response (positive response to anti-CTLA4 and anti-PDCD1 therapy) than the high CRLnc risk group. Although the drug sensitivity analysis revealed that there were no significant differences in the IC_50_ of various antitumor drugs between the high- and low-risk groups, these findings may support the selection of clinical medication for the treatment of HCC.

Considering the stability of lncRNAs in body fluids and their potential for tumor typing (11, 40), we constructed three CRLncC and CRGCs based on the CRLnc and cuproptosis-related differentially expressed genes to better understand the cuproptosis-related patterns. The results showed that the CRGC was closely correlated with clinicopathological features, prognosis, and the TME of HCC compared with the CRLncC. According to the relationship between the immune response process and molecular classification of the TME in HCC, three immune clusters were classified, namely, the immune-high, immune-mid, and immune-low clusters. The immune-high cluster was characterized by increased B-cell, plasma cell, and T-cell infiltration, with variable increases observed in other immune cell types. The immune-mid cluster was characterized by increased infiltration of T cells and other immune cells, with lesser B-cell and plasma cell infiltration. The immune-low cluster was characterized by the lowest infiltration by immune cells (39, 41). CRGC-B exhibited the lowest tumor purity and increase in stromal, immune, and ESTIMATE scores. We also found that CRGC-B was closely linked to better clinical features and prognosis in patients with HCC than the other two clusters. Additionally, CRGC-B was more prone to immune-high cluster, with greater abundance of T cells, dendritic cells, natural killer cells, and B cells, and was more likely to respond to treatment with ICIs. Surprisingly, there was a large number of macrophages in all three CRGCs. Tumor-associated macrophages have been associated with antitumor activity (M1-like tumor-associated macrophages) as well as immunosuppressive and tumor-promoting effects (M2-like) (42, 43). Hence, further research is needed to explore the role of cuproptosis in the TME. By analyzing the biological processes and immune microenvironments of different subtypes, it was revealed that different CRGCs have distinctive clinical and immune characteristics. This observation indicated that such typing may be applicable to the management of HCC.

The univariate Cox proportional hazards regression model and risk results showed that lncRNA AL158166.1 had the highest hazard ratio among those examined. Thus far, the role of AL158166.1 in tumor cells has not been studied *in vitro*. We found that the levels of AL158166.1 were higher in HCC tissues versus healthy tissues. Knockdown of AL158166.1 suppressed the proliferation and migration of HCC cells, whereas the OV of AL158166.1 exerted the reverse effect. Knocking down of lncRNA AL158166.1 could induce an increase in Cu accumulation in HCC cells, decrease the expression of DLAT, and inhibit the HCC cell growth. Cu2+ could bind to thioacylated DLAT, inducing the isomerization of DLAT. The increase in insoluble DLAT leads to cytotoxicity and induces cell death (34). These findings showed that AL158166.1 may be a target for the prediction of prognosis and treatment of HCC.

This study had several limitations. Firstly, all data were obtained from TGCA database and retrospectively analyzed; hence, inherent bias may be present in the interpretation of the results. Secondly, considering the nature of this study, large-scale prospective research, as well as *in vivo* and *in vitro* experiments, is warranted to verify the present findings. Lastly, there was a relative lack of clinical information, including the use of neoadjuvant chemotherapy and chemoradiotherapy.

## 5 Conclusion

The new CRLncSig prognostic risk model may predict the prognosis of HCC and response to immunotherapy. Based on the identification of immune types for individualized therapy, CRGs and CRLnc will hopefully overcome systemic treatment failure and broaden the applicability of immunotherapy. Therefore, further research is warranted to elucidate the mechanism of cuproptosis and its relationship with lncRNAs and the TME in HCC. This prognostic risk model and cuproptosis-related molecular signature may provide a new idea for cuproptosis-related clinical features and the treatment of HCC.

## Data availability statement

The original contributions presented in the study are included in the article/**Supplementary Material**. Further inquiries can be directed to the corresponding authors.

## Ethics statement

The studies involving humans were approved by the ethics review committee/Shanghai General Hospital. The studies were conducted in accordance with the local legislation and institutional requirements. The human samples used in this study were acquired from primarily isolated as part of your previous study for which ethical approval was obtained. Written informed consent for participation was not required from the participants or the participants’ legal guardians/next of kin in accordance with the national legislation and institutional requirements.

## Author contributions

JX and SL designed the study. ZZ and JW conducted the experiments and analyzed the data. JL, WC, FS, PZ, and CX supervised and proofed the data analysis. SL, JW, and ZZ wrote and proofed the manuscript. SL, JW, and JL contributed equally. All authors contributed to the article and approved the submitted version.

## Supplementary material

Figure S1 The entire analytical process of the study.

Figure S2
**(A)** Correlation between 16 cuproptosis related genes and 6 model lncRNAs. **(B)** The heatmap of 6 model lncRNAs expression and clinicopathological features in high and low risk group. **(C–E)** The enrichment of immune cells and immune functions in genecluster-A, B, and C.

Figure S3
**(A–C)** Consensus matrix heatmap defining three cuproptosis related lncRNAs clusters (k=3) and their correlation area. **(D)** PCA analysis for three cuproptosis related lncRNAs clusters to distinguish samples in TCGA HCC. **(E)** Differences in risk scores between three lncRNAs subtypes. **(F)** Survival analyses for three lncRNAs clusters using Kaplan-Meier curves (P=0.003). **(G)** Survival analyses for lncRNAs cluster **(B, C)** using Kaplan-Meier curves (P<0.132). **(H–K)** The tumor purity, immune, stromal and ESTIMATE score in three lncRNAs clusters by ESTIMATE algorithm.
