# Supplementary figures and images for: Prediction of prognosis, immune infiltration, and personalized treatment of hepatocellular carcinoma by analysis of cuproptosis-related long noncoding RNAs and verification in vitro
Source: Front Oncol. 2023 Sep 8;13:1159126. doi: 10.3389/fonc.2023.1159126 (PMC10514553; doi:10.3389/fonc.2023.1159126)

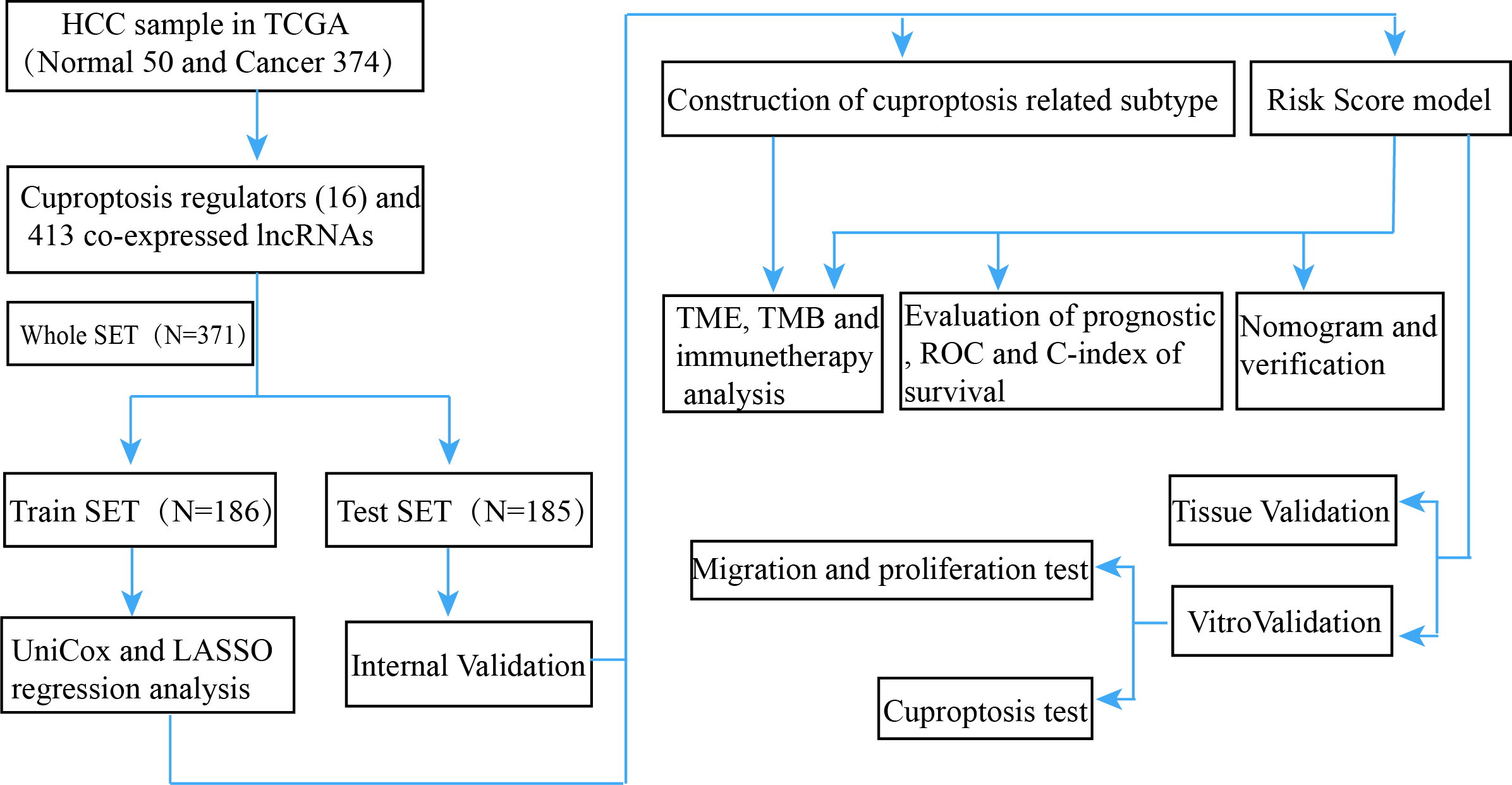

Supplement: Figure S1 — The entire analytical process of the study. [file Image_1.tif]

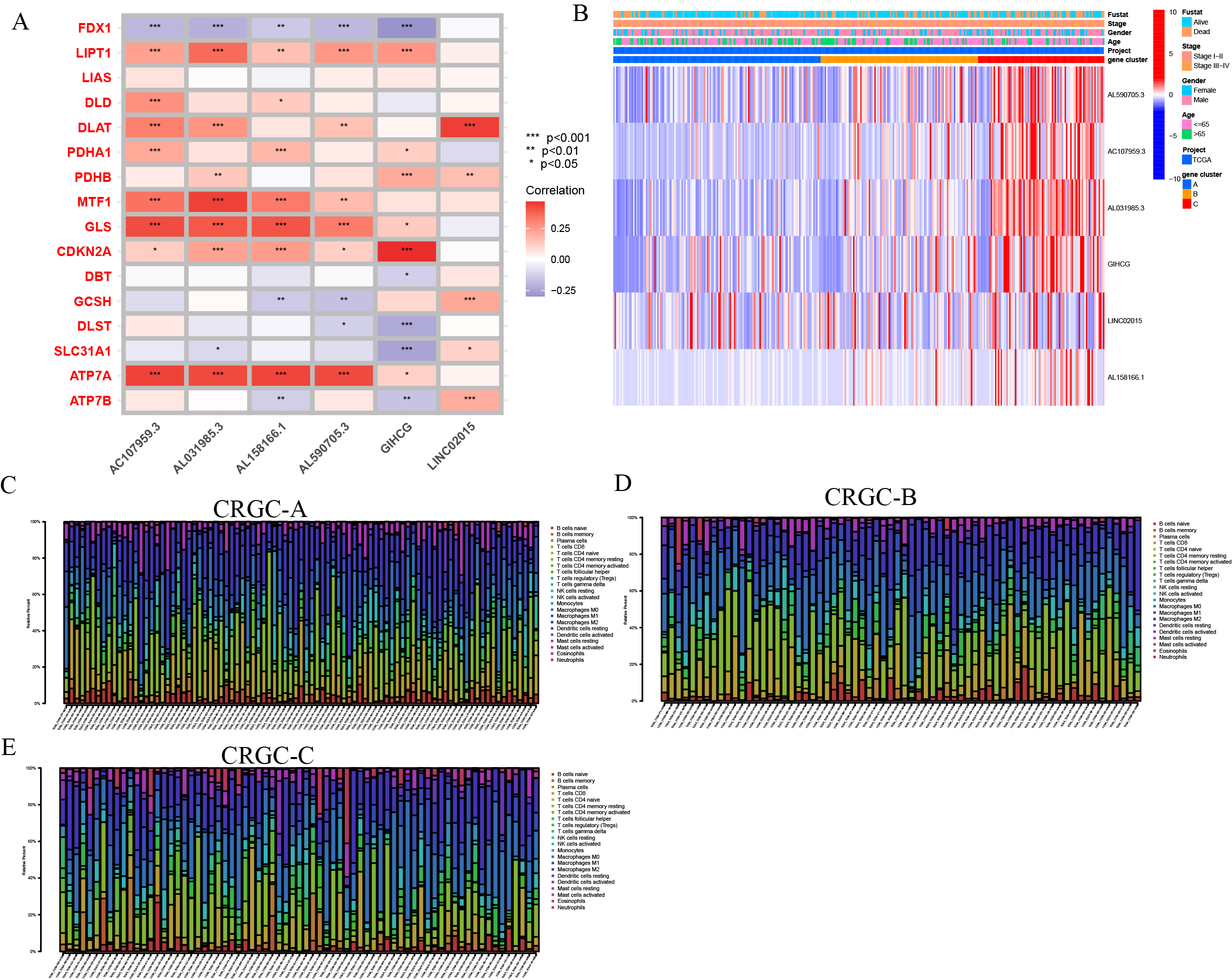

Supplement: Figure S2 — (A) Correlation between 16 cuproptosis related genes and 6 model lncRNAs. (B) The heatmap of 6 model lncRNAs expression and clinicopathological features in high and low risk group. (C–E) The enrichment of immune cells and immune functions in genecluster-A, B, and C. [file Image_2.tif]

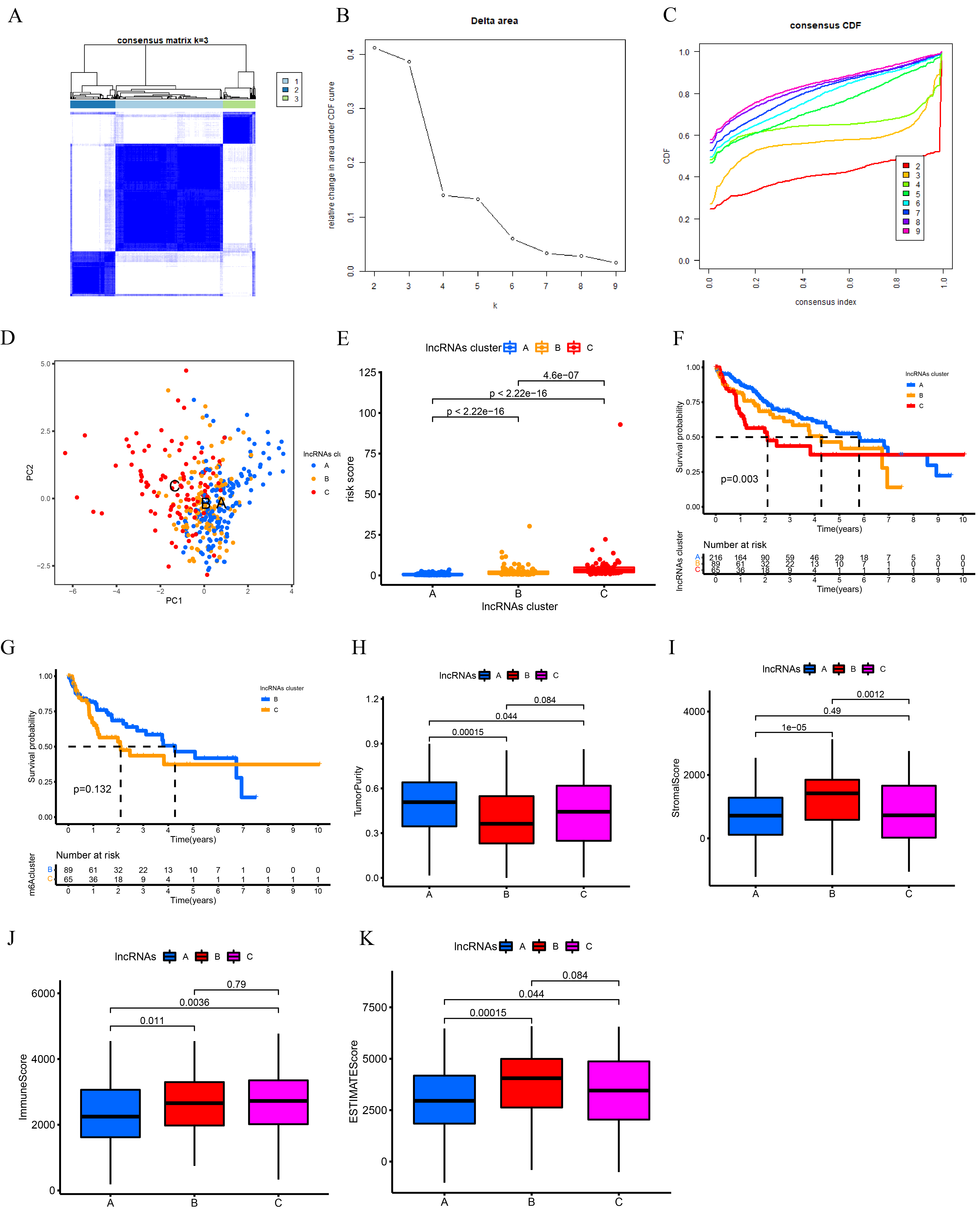

Supplement: Figure S3 — (A–C) Consensus matrix heatmap defining three cuproptosis related lncRNAs clusters (k=3) and their correlation area. (D) PCA analysis for three cuproptosis related lncRNAs clusters to distinguish samples in TCGA HCC. (E) Differences in risk scores between three lncRNAs subtypes. (F) Survival analyses for three lncRNAs clusters using Kaplan-Meier curves (P=0.003). (G) Survival analyses for lncRNAs cluster (B, C) using Kaplan-Meier curves (P<0.132). (H–K) The tumor purity, immune, stromal and ESTIMATE score in three lncRNAs clusters by ESTIMATE algorithm. [file Image_3.tif]
